# Variability and social patterning of cancer mortality in 343 Latin American cities: an ecological study

**DOI:** 10.1016/S2214-109X(24)00446-7

**Published:** 2025-01-29

**Authors:** Tania Alfaro, Kevin Martinez-Folgar, Dalia Stern, Maria A Wilches-Mogollon, María Pía Muñoz, Harrison Quick, Marcio Alazraqui, Manuel Ramirez-Zea, J Jaime Miranda, Mariana Lazo, Waleska Teixeira Caiaffa, Ana V Diez Roux, Usama Bilal

**Affiliations:** a Programa de Doctorado en Salud Pública, Escuela de Salud Pública, Facultad de Medicina, Universidad de Chile, Santiago, Chile; b Center for Global Health Equity, University of Michigan, Ann Arbor, MI, USA; c Centro de Investigación del INCAP para la Prevención de Enfermedades Crónicas (CIIPEC), Instituto de Nutrición de Centro América y Panamá, Guatemala City, Guatemala; d CONAHCyT-Centro de Investigación en Salud Poblacional, Instituto Nacional de Salud Pública (INSP), Cuernavaca, Mexico; e School of Medicine, Universidad de Los Andes, Bogotá, Colombia; f Escuela de Salud Pública, Facultad de Medicina, Universidad de Chile, Santiago, Chile; g Urban Health Collaborative, Dornsife School of Public Health, Drexel University, Philadelphia, PA, USA; h Department of Community Health and Prevention, Dornsife School of Public Health, Drexel University, Philadelphia, PA, USA; i Department of Epidemiology and Biostatistics, Dornsife School of Public Health, Drexel University, Philadelphia, PA, USA; j Instituto de Salud Colectiva - Universidad Nacional de Lanús, Buenos Aires, Argentina; k CRONICAS Centre of Excellence in Chronic Diseases, Universidad Peruana Cayetano Heredia, Lima, Peru; l School of Medicine, Universidad Peruana Cayetano Heredia, Lima, Peru; m Observatory for Urban Health in Belo Horizonte, Federal University of Minas Gerais, Belo Horizonte, Brazil

## Abstract

**Background:**

Understanding between-city variations in cancer mortality is crucial to inform national and subnational cancer prevention strategies. However, studies at the city level in Latin America are scarce. As part of the Salud Urbana en América Latina (SALURBAL) project, we aimed to describe the variability in cancer mortality rates across 343 cities in nine Latin American countries and the associations of these rates with city-level socioeconomic development.

**Methods:**

This ecological study used data from cities in Argentina, Brazil, Chile, Colombia, Costa Rica, El Salvador, Guatemala, Mexico, and Panama. Vital registration and population data from Jan 1, 2015 to Dec 31, 2019 were used to estimate sex-specific and age-standardised cancer mortality rates for each city, overall and for seven cancer sites (breast, lung, colorectal, stomach, liver, prostate, and cervical), and the associations of these rates with city-level socioeconomic development.

**Findings:**

We found wide variability in cancer mortality by city (overall age-adjusted cancer mortality rates varied by almost three times), sex, and cancer site. Variability between cities within the same country was highest for cervical and prostate cancer. The most common causes of cancer deaths were breast cancer (305 cities) for females and prostate cancer (167 cities) and lung cancer (132 cities) for males. Liver and cervical cancer were the primary cause of cancer mortality in fewer than ten cities each, most of which were in Guatemala and Mexico. Lower city-level socioeconomic development was associated with higher mortality from liver, stomach, cervical, and prostate cancers and lower mortality from breast, colorectal, and lung cancers, with variations by sex.

**Interpretation:**

We found considerable heterogeneity in cancer mortality between cities, geographical patterning, and associations between cancer mortality rates and socioeconomic development. Our results highlight the need to consider city contexts when planning interventions to reduce cancer mortality and when guiding future cancer prevention and control efforts in urban areas within the region.

**Funding:**

Wellcome Trust.

**Translations:**

For the Spanish and Portuguese translations of the abstract see Supplementary Materials section.

## Introduction

According to the International Agency for Research on Cancer, there were approximately 10 million deaths from cancer in 2022 and 20 million new cases of cancer,^1^ 70% of which were in low-income and middle-income countries (LMICs).^2^ Data such as those from the International Agency for Research on Cancer show that, in contrast to most high-income countries, cancer mortality rates in LMICs are increasing or stagnating^2^ and the incidence of cancer is increasing.^2^, ^3^ In Latin America, there were around 1·5 million new cases of cancer and 750 000 deaths from cancer in 2022.^1^ The most common cancers in Latin America include both lifestyle-related (eg, breast and lung) and infection-related (eg, stomach and cervical) cancers.^4^ Geographical differences in cancer incidence, survival, and mortality have been observed at the country level in the region;^4^, ^5^ however, variations across smaller areas have been less well explored.

Incidence trends in LMICs and Latin America have been attributed to ageing, population growth, and lifestyle changes. Known risk factors for cancer (eg, obesity, unhealthy diet, physical inactivity, smoking, and excessive alcohol consumption)^2^ are linked to socioeconomic development and urbanisation.^1^, ^2^, ^4^ Latin America has had a rapid urbanisation process over the past 70 years and has become one of the most urbanised regions worldwide, with 80% of the population of this region now living in cities.^6^, ^7^ Urbanisation and development patterns vary, as do their effects on exposures that are specifically linked to cancer; as such, understanding the cancer burden in these cities is essential.

Urbanisation could contribute to increased rates of cancers linked to behavioural changes such as more sedentary lifestyles, higher consumption of ultra-processed foods, increased smoking and alcohol consumption, and greater exposure to pollutants.^2^, ^4^, ^8^ However, urbanisation also offers increased opportunities for the control (ie, prevention, detection, and treatment) of cancer.^9^ City policies and urban design can promote healthy lifestyles, decrease exposure to carcinogenic factors such as air pollution, and facilitate protective factors such as green spaces.^2^ Well planned cities could facilitate health-care use, improving access to preventive care and appropriate specialised cancer care, whereas socioeconomic inequality could hamper these efforts.^10^ Here we aim to describe the variability in cancer mortality across 343 Latin American cities spanning nine countries and investigate the associations between cancer mortality rates and socioeconomic development.

**Research in context**

**Evidence before this study**

Cancer is the second leading cause of death worldwide. Latin America, one of the world's most urbanised regions, has a double burden of lifestyle-related and infection-related cancers, both of which are potentially associated with diverging levels of socioeconomic development. We searched PubMed and SciELO from database inception to Sept 25, 2020—later updating the search to Dec 6, 2024—for records published in English or Spanish using the search terms (“cancer” OR “neoplasms”) AND (“mortality”) AND (“Latin America”) AND (“city” or “cities”). We identified 155 unique articles, 11 of which were local studies focused on cities (nine) or larger geographical regions (two) in Argentina, Brazil, Colombia, Ecuador, and Peru. To our knowledge, there has been no subnational (ie, city-level) characterisation of cancer mortality across a large sample of urban areas in the region and its variation with socioeconomic development.

**Added value of this study**

To our knowledge, this study is the first to report cancer mortality and its associations with socioeconomic development, at the city level, for a large number of Latin American cities. We analysed data from 343 cities from 2015 to 2019 and found substantial heterogeneity between cities, with overall age-adjusted cancer mortality rates varying almost three-fold. For some cancers, such as cervical cancer in females and prostate cancer in males, we observed higher variability between cities within the same country than between countries. Although liver cancer was rare in most of the region, it was a common cause of cancer mortality in some cities in Mexico and Guatemala, highlighting context-specific risk factors in the region. We found that cervical, liver, prostate, and stomach cancer mortality rates were higher in cities with lower levels of socioeconomic development, whereas breast, colorectal, and lung cancer mortality rates were higher in cities with higher levels of socioeconomic development.

**Implications of all the available evidence**

Considering the rapid urbanisation that has been occurring in Latin America over the past 70 years, these results could help to inform the development of policies for the prevention, detection, and treatment of cancer at the city level and to identify topics on which additional research is needed.

## Methods

### Study design

This ecological study was part of the Salud Urbana en América Latina (SALURBAL) project,^7^, ^11^ which compiled and harmonised mortality, social, and built environment data from 371 cities with at least 100 000 inhabitants in 11 Latin American countries (details on the selection and definition of cities are available elsewhere^7^ and in appendix 3 [pp 2–7]). Each city was defined as urban agglomerations of administrative units (eg, *municipios* or *comunas*) that overlapped with the urban extent of the city. We included all 343 cities in nine countries (Argentina, Brazil, Chile, Colombia, Costa Rica, El Salvador, Guatemala, Mexico, and Panama) for which population and mortality data were available at the city level at the time of the study. Mortality data—age, sex (assigned at birth), and underlying cause of death (coded using ICD-10)—were obtained from universal vital registration systems in each country for the 2015–19 period.^11^ Deaths in all age groups were considered. We obtained population denominators for the same period and social environment variables based on census data from the SALURBAL study. Details of the data sources are available elsewhere.^7^, ^11^

The SALURBAL study protocol was approved by the Drexel University Institutional Review Board (ID number 1612005035).

### Procedures

We classified deaths with ICD-10 codes C00–C97 as attributed to cancer. We selected the five most common anatomical sites for cancer (both sexes pooled) on the basis of their burden in Latin America: trachea, bronchus, and lung (henceforth lung); stomach; colon and rectum (henceforth colorectal); breast; and prostate. We added two anatomical sites for cancer that are particularly relevant in central America: cervix uteri (henceforth cervical) and liver.^4^ Specific ICD-10 codes for each anatomical site are detailed in appendix 3 (p 8). We used two categorisations for analytical purposes: overall cancer deaths (all cancer sites including non-melanoma skin cancer) and site-specific deaths. To address three important issues with vital registration data, we imputed missing age in approximately 0·5% of records and missing sex in approximately 0·2% of records using a proportional redistribution by age, sex, cause of death, country, and year; corrected for the incomplete coverage of all deaths; and redistributed deaths categorised as ill-defined. The three approaches we used are detailed elsewhere^11^ and descriptive statistics of coverage and ill-defined deaths are shown in appendix 3 (p 9).

### Exposures

We used a comprehensive social environment index (SEI) developed by Bilal and colleagues^11^ as a proxy for city-level socioeconomic development. We summed the Z scores of indicators of education (proportion of the population aged 25 years or older that completed primary education), overcrowding (percentage of households with more than three people per room, reversed), and water and sanitation access (proportion of houses with piped water access inside the dwelling and connection to public sewage network). This information was obtained from the last national census available for each country for which data on these indicators were recorded (appendix 3 p 10). We divided the SEI into quartiles, where quartile 1 (−2·03 to −0·25) was defined as low SEI, quartile 2 (>−0·25 to 0·17) was defined as mid–low SEI, quartile 3 (>0·17 to 0·51) was defined as mid–high SEI, and quartile 4 (>0·51 to 1·09) was defined as high SEI. A higher SEI is a proxy for better social conditions.^11^

### Statistical analysis

First, we computed age-specific and sex-specific overall and anatomical site-specific cancer mortality rates for each city for 2015–19 and used these rates to compute age-standardised cancer mortality rates using the WHO 2000–25 standard population. All rates were reported per 100 000 population. Second, we ranked site-specific cancer mortality rates overall by quartiles of the SEI for all cities combined. We also computed the number of cities for which each cancer site was between the first and the sixth most common cause of cancer death. Third, to describe variability in cancer mortality rates across cities and countries, we calculated intraclass correlation coefficients using an intercept-only linear mixed model, with the log-transformed mortality rate as the outcome and a normally distributed random intercept for each country.

Finally, we explored the association between city-level SEI and cancer mortality rates overall and for seven selected anatomical cancer sites, using a multilevel negative binomial model stratified by sex with one observation per city-age group, the log(count) of deaths as the outcome, a normally distributed random intercept for each country, a fixed effect for age group (0–14, 15–39, 40–54, 55–64, 65–74, and ≥75 years) and for the SEI, and an offset for sex-specific population. To assess whether associations differed by sex, we fitted a model with both sexes pooled and an interaction coefficient between the SEI and sex. We also used results from this model to calculate a measure of additive interaction (the relative excess risk due to interaction [RERI]^12^), for which we dichotomised the SEI to avoid having three different RERI estimates per outcome, and also reverse-coded the SEI for the three sites (overall, liver, and stomach) for which there was a negative association between SEI and mortality, as the RERI can be consistently estimated only for positive associations.^12^ To facilitate interpretation, SEI in all models was categorised into quartiles. We also fitted a model using restricted cubic splines with three knots (at the 25th, 50th, and 75th percentiles). Analyses were conducted in R version 4.1 using the glmmTMB package.

### Role of the funding source

The funder of the study had no role in study design, data collection, data analysis, data interpretation, or writing of the report.

## Results

**Figure 1:**
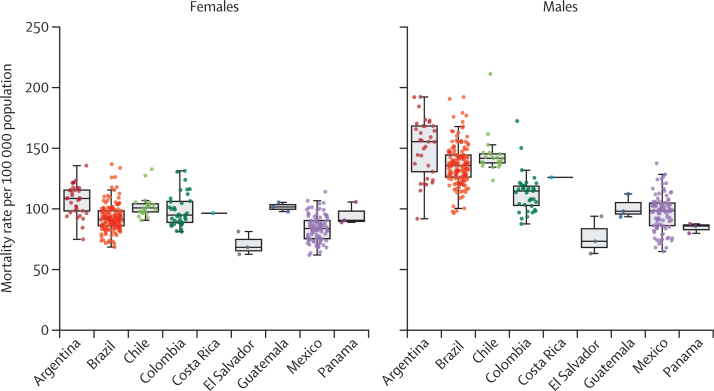
Age-standardised cancer mortality rates, stratified by sex The middle line represents the median city cancer mortality rate, the box limits represent the 25th and 75th percentiles, and the whiskers represent 1·5 times the IQR. Individual datapoints (cities) are shown as dots.

Between Jan 1, 2015 and Dec 31, 2019, 1 567 663 cancer deaths occurred in the 343 cities in our study, accounting for 17·6% of all-cause mortality. The overall cancer mortality rate was 109·9 per 100 000 population: 113·1 for males and 107·1 for females. The overall age-standardised cancer mortality rates, stratified by country and sex, are shown in figure 1. We found wide variability in cancer mortality rates between cities, with 53% of the total overall cancer mortality variability occurring within countries for females and 26% for males (appendix 3 p 11). For males, the city with the highest overall cancer mortality rate was Antofagasta, Chile (211·3 per 100 000 males) and the city with the lowest rate was San Miguel, El Salvador (63·2 per 100 000 males). For females, the highest overall cancer mortality rate was in Rio Grande, Brazil (136·9 per 100 000 females) and the lowest was in Tecoman, Mexico (61·9 per 100 000 females).

**Figure 2:**
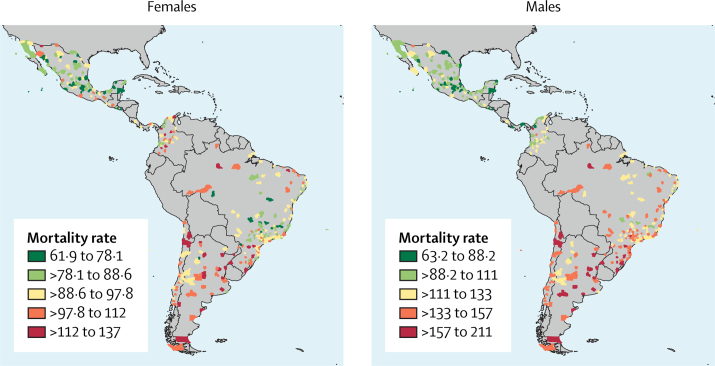
Geographical distribution of overall age-standardised cancer mortality rates, by sex and city

Figure 2 shows the geographical distribution of overall age-standardised cancer mortality rates by sex and city. We found higher rates for both males and females in South America than in central America and Mexico. Among females, we observed some of the highest rates in the north of Mexico and in some Colombian cities, and most cities in Brazil had lower rates than other cities in South America.

Age-adjusted mortality rates by cancer site are shown in appendix 3 (pp 12, 19). We found wide variability between cities within countries, although the variability differed by site and sex. For females, the variability in cervical cancer rates was 79% between cities within countries; for the other cancer sites, this value ranged from 34% to 45% (appendix 3 p 11). In males, prostate cancer had a much higher variability between cities within countries (66%) than the other cancer sites (32–40%). For male and female sexes combined, the five leading causes of cancer mortality across all cities were lung, colorectal, prostate, stomach, and breast cancer; separated by sex, the leading causes were prostate, lung, stomach, colorectal, and liver cancer for males and breast, lung, colorectal, cervical, and stomach cancer for females. Lung cancer was the leading cause of cancer mortality (males and females combined) in 214 of 343 cities, with 186 480 deaths and a mortality rate of 13·1 per 100 000 population. However, wide variations were observed between cities (ranging from 39·9 in Antofagasta, Chile to 3·2 in Tianguistenco, Mexico). By sex, lung cancer was the leading cause of cancer mortality in 18 of 343 cities for females and 132 of 343 cities for males (appendix 3 pp 12–13).

**Figure 3:**
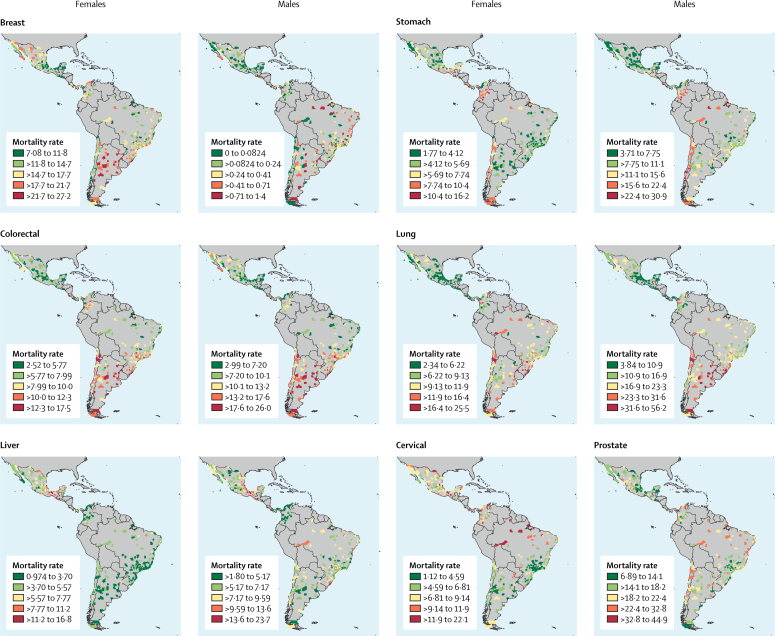
Geographical distribution of age-standardised cancer mortality rates for seven cancers, by sex and city

Figure 3 shows the geographical distribution of site-specific cancer mortality rates by city and sex. For females, a higher mortality rate was observed for cervical and liver cancer in Mexico and central America than in South America. We also found some cities with high cervical cancer mortality rates in Brazil and in the north of Argentina. Colorectal and breast cancer mortality rates were especially high in South America. High stomach cancer mortality rates were observed in central and South America. For males, the highest lung, colorectal, and stomach cancer mortality rates were concentrated in South America. The highest liver cancer mortality rates were observed primarily in Mexican and central American cities. For stomach cancer, especially in males, high mortality rates were found in Andean cities—specifically in Colombia and Chile.

Table 1 ranks the site-specific cancer mortality rates for all cities, by quartiles of SEI, stratified by sex. The same ranking, including the largest cities in each country and the position of the seven selected sites in the ranking among all cancer sites, is shown in appendix 3 (pp 14–15). For females, breast cancer was the most common cause of cancer death across all SEI quartiles. However, the next most common cancer sites varied with SEI, with cervical cancer being second in cities with low SEI and lung and colorectal cancer being second and third in all other cities. For males, we found more consistent patterns by SEI: prostate cancer was the most common cause of cancer death among low SEI and mid–low SEI cities, followed by lung cancer, whereas lung cancer was the most common cause of cancer death in mid–high SEI and high SEI cities, followed by prostate cancer. By city, patterns were more heterogeneous.

**Table 1:** Ranking of the seven age-standardised, site-specific cancer mortality rates by SEI quartile, stratified by sex

	**Breast**	**Cervical**	**Colorectal**	**Liver**	**Prostate**	**Stomach**	**Lung**
**Females**							
Overall	1	4	3	6	NA	5	2
Quartile 1: low SEI	1	2	4	5	NA	6	3
Quartile 2: mid–low SEI	1	4	3	6	NA	5	2
Quartile 3: mid–high SEI	1	4	2	6	NA	5	3
Quartile 4: high SEI	1	5	3	6	NA	4	2
**Males**							
Overall	NA	NA	4	5	1	3	2
Quartile 1: low SEI	NA	NA	5	4	1	3	2
Quartile 2: mid–low SEI	NA	NA	3	5	1	4	2
Quartile 3: mid–high SEI	NA	NA	3	5	2	4	1
Quartile 4: high SEI	NA	NA	3	5	2	4	1

Ranking is from 1 to 6, with 1 being the leading cause of cancer mortality. SEI quartiles are defined as follows: quartile 1 −2·03 to −0·25; quartile 2 >–0·25 to 0·17; quartile 3 >0·17 to 0·51; quartile 4 >0·51 to 1·09. NA=not applicable. SEI=social environment index.

Table 2 shows the results of regression modelling exploring the associations between cancer mortality and city-level SEI, stratified by sex. We found no evidence of associations between city-level SEI and overall cancer mortality rates for females; however, in almost all cancer sites, associations with SEI followed a dose–response pattern. In cities with high SEI, the mortality rate of cervical cancer was 44% (95% CI 40–48) lower than in cities with low SEI; analogous values were 30% (26–35) for liver cancer and 17% (12–22) for stomach cancer. Conversely, mortality rates of breast cancer were 15% (10–19) higher, colorectal cancer were 33% (27–39) higher, and lung cancer were 13% (6–20) higher in cities with high SEI than in cities with low SEI.

**Table 2:** Mortality rate ratios for each cancer site by SEI, stratified by sex

	**Overall**	**Breast**	**Cervical**	**Colorectal**	**Liver**	**Stomach**	**Lung**	**Prostate**
**Females**								
Quartile 1: low SEI	1 (ref)	1 (ref)	1 (ref)	1 (ref)	1 (ref)	1 (ref)	1 (ref)	NA*
Quartile 2: mid–low SEI	1·01 (0·99–1·03)	1·12 (1·07–1·16)	0·75 (0·71–0·80)	1·18 (1·13–1·24)	0·81 (0·76–0·86)	0·90 (0·85–0·95)	1·05 (0·99–1·11)	NA*
Quartile 3: mid–high SEI	0·99 (0·97–1·01)	1·13 (1·09–1·18)	0·62 (0·58–0·66)	1·30 (1·24–1·35)	0·73 (0·69–0·78)	0·86 (0·81–0·91)	1·06 (1.00–1·12)	NA*
Quartile 4: high SEI	1·00 (0·97–1·02)	1·15 (1·10–1·19)	0·56 (0·52–0·60)	1·33 (1·27–1·39)	0·70 (0·65–0·74)	0·83 (0·78–0·88)	1·13 (1·06–1·20)	NA*
**Males**								
Quartile 1: low SEI	1 (ref)	NA*	NA*	1 (ref)	1 (ref)	1 (ref)	1 (ref)	1 (ref)
Quartile 2: mid–low SEI	1·06 (1·03–1·09)	NA*	NA*	1·25 (1·19–1·32)	0·87 (0·82–0·93)	0·91 (0·87–0·97)	1·17 (1·11–1·24)	0·96 (0·92–1·01)
Quartile 3: mid–high SEI	1·04 (1·02–1·07)	NA*	NA*	1·43 (1·36–1·50)	0·85 (0·80–0·90)	0·90 (0·85–0·95)	1·18 (1·11–1·25)	0·90 (0·86–0·94)
Quartile 4: high SEI	1·09 (1·06–1·12)	NA*	NA*	1·54 (1·46–1·62)	0·87 (0·81–0·92)	0·89 (0·84–0·94)	1·22 (1·14–1·29)	0·90 (0·86–0·94)

Data are exponentiated coefficients and 95% CI. We used age-adjusted negative binomial models, with a random intercept for each country and a fixed effect for age group. SEI quartiles are defined as follows: quartile 1 −2·03 to −0·25; quartile 2 >–0·25 to 0·17; quartile 3 >0·17 to 0·51; quartile 4 >0·51 to 1·09. p values for each coefficient are provided in appendix 3 (p 16). NA=not applicable. SEI=social environment index.

* Data on breast cancer and cervical cancer for males and prostate cancer for females were too sparse, leading to model non-convergence.

In males, we found an association between city-level SEI and overall cancer mortality rates: in cities with high SEI, rates were 9% (95% CI 6–12) higher than in cities with low SEI. Considering specific cancer sites, rates of liver cancer were 13% (95% CI 8–19) lower, stomach cancer were 11% (6–16) lower, and prostate cancer were 10% (6–14) lower in cities with high SEI than in cities with low SEI. The association between higher SEI and lower liver and stomach cancer rates was stronger for females than for males on both the multiplicative and additive scales (appendix 3 pp 17–18). Similarly to females, in males, mortality rates of colorectal cancer were 54% (46–62) higher and lung cancer were 22% (14–29) higher in cities with high SEI than in cities with low SEI; both associations were stronger for males than for females.

The variability in proportionate cancer mortality by site and SEI (p 20) and the association between cancer mortality by site and continuous SEI (p 21) are shown in appendix 3. We found higher proportions of lung, colorectal, and breast cancer in cities with higher SEI than those with lower SEI, and higher proportions of liver and cervical cancer in cities with lower SEI. We also found some non-linearities at the lower end of the SEI distribution (where the number of cities is sparse) for prostate and stomach cancer.

## Discussion

Our study of more than 1·5 million cancer deaths in 343 cities in nine Latin American countries from 2015 to 2019 revealed three key findings. First, we found substantial heterogeneity in cancer mortality rates across cities, with overall age-adjusted rates varying almost threefold. However, this heterogeneity varied widely by cancer site, with cervical cancer among females and prostate cancer among males varying much more between cities within countries than between countries. Second, whereas breast cancer was the most frequent cause of cancer mortality in females, prostate and lung cancer were the most common for males. Notably, although deaths from liver cancer were rare in most of Latin America, this cancer was a common cause of cancer mortality in some cities in Mexico and Guatemala, highlighting context-specific risk factors in the region. Finally, although we did not find associations between city-level socioeconomic development and overall cancer mortality, we found that cervical, liver, prostate, and stomach cancer mortality rates were higher in cities with a lower SEI, whereas breast, colorectal, and lung cancer mortality rates were higher in cities with a higher SEI. These associations varied by sex, with cancers linked to lower socioeconomic development having stronger associations in women and cancers linked to higher socioeconomic development having stronger associations in men.

We found an almost three-fold difference in the overall cancer mortality rate when comparing cities with the highest rates to those with the lowest rates. This result follows previous findings showing large variability between city-level life expectancy and proportionate mortality.^11^ This finding was especially notable for cervical cancer (79% of variability was within countries) and prostate cancer (66%). In the case of cervical cancer, a preventable neoplasm for which effective screening and vaccination are available, studies in the USA and China have shown differences in screening coverage according to geography.^13^, ^14^ To our knowledge, such studies are still scarce in Latin America. Although the ability to prevent prostate cancer through screening is less clear than for cervical cancer, previous studies have found higher proportions of testing and better access to health care in urban than in rural areas of Latin America.^15^ These results are consistent with a previous study in which we found that variability in mortality due to conditions—including cervical cancer—for which health systems could prevent incident cases was higher within countries than between countries, especially for females.^10^ We also found that, for cancer sites for which we could compare rates between sexes (ie, lung, colorectal, stomach, and liver), variability within countries was consistently higher for females than for males, especially in the case of lung cancer (44% within-country variability for females and 32% for males). This finding indicates that the distribution of drivers of lung cancer mortality (and, to a lesser extent, colorectal and liver cancer mortality) varies more within countries for females than for males. In summary, our findings showed differences in cancer mortality rates across cities, suggesting that city policies and urban design could offer an opportunity for cancer control; indeed, initiatives such as City Cancer Challenge have considered cities as the focus of interventions to improve cancer care.^9^ These factors should be taken into account in the Latin America and the Caribbean Code Against Cancer.^16^

In this study, we found that breast, lung, colorectal, cervical, and stomach cancer were the most common causes of cancer death among females, and prostate, lung, stomach, colorectal, and liver cancer were the most common among males, matching International Agency for Research on Cancer data for Latin America and the Caribbean region for 2022. However, we found variations in these rankings by city-level socioeconomic development levels and for a sample of large cities in the region, pointing to the importance of considering local context in cancer control. Latin America has experienced a rapid epidemiological transition driven by population ageing, lifestyle changes, and urbanisation.^17^ This transition has been heterogeneous between and within countries^11^ and has been influenced by various factors, including marked inequities.^6^, ^17^ Assuming a homogeneous epidemiological transition across cities of the region could lead to inadequate recommendations regarding priority targets for cancer control.

As an example of the heterogeneity across cities, we consider liver cancer, for which we observed higher mortality in some cities of Guatemala and southern Mexico. Whereas liver cancer was the eighth (in females) and the sixth (in males) most common cause of cancer death across all sites, it was the first or second most common among the cancer sites we studied in 17 cities in females and in 22 cities in males—mostly in Guatemala and Mexico. Liver cancer is caused by non-infectious risk factors (eg, obesity, alcohol, and fatty liver disease), viral hepatitis (after infection with hepatitis viruses), and exposure to aflatoxin.^18^ A common contaminant of corn, which is consumed as a dietary staple, aflatoxin is particularly prevalent in Guatemala and southern Mexico—especially in poorer rural areas but also in urban environments.^18^, ^19^ Urban environments in the region also have a combination of other risk factors for liver cancer—in particular alcohol consumption, obesity, and diabetes.^19^ This combination of a disease associated with lower levels of socioeconomic development and increased exposure to risk factors in urban environments (eg, ultra-processed foods and alcohol marketing) creates an ideal risk environment for an increased burden of liver cancer. Our study suggests that liver cancer control should be a public health priority for some cities in the region, emphasising the need for appropriate targeting in the efforts to control risk factors and improve early diagnosis and treatment in Latin America.

Finally, we explored associations between city-level socioeconomic development and cancer mortality rates. Two key findings emerged from this analysis. First, although associations with overall cancer mortality rates were non-existent or weak, associations with site-specific cancer mortality rates were strong. We found two distinct clusters: one linked to lower levels of socioeconomic development (liver and stomach cancer in both males and females, cervical cancer in females, and prostate cancer in males) and one linked to higher levels of socioeconomic development (colorectal and lung cancer in both males and females and breast cancer in females). These findings are consistent with other studies in Latin America, including one examining breast and cervical cancer in Brazil.^20^ Cervical, liver, and stomach cancers have important infectious risk factors that follow patterns similar to those of other communicable diseases, the incidence of which decreases as city-level socioeconomic development increases.^11^ Conversely, breast and colorectal cancers, which are associated with higher socioeconomic development, have shown an increasing trend in various Latin American countries.^4^

We found that the association of city-level socioeconomic development with cancer mortality systematically varied by sex. The cancer sites associated with lower levels of socioeconomic development had stronger negative associations in females than in males. Conversely, for cancers associated with higher levels of socioeconomic development, the positive associations were stronger in males than in females. An example of this effect modification by sex can be found in lung cancer: for females, compared with cities with low SEI, cities with mid–low SEI have 5% higher mortality, those with mid–high SEI have 6% higher mortality, and those with high SEI have 13% higher mortality; for males, these numbers increase to 17%, 18%, and 22%, respectively. The main risk factor for lung cancer is smoking, with some contributions from air pollution.^21^ Considering the stages of the smoking epidemic theory (which shows sequential smoking adoption and cessation by males followed by females),^22^ the relationship between gender equality and gender smoking patterns,^23^ the lags between smoking and lung cancer mortality,^24^ and the heterogeneity in lung cancer mortality rates between cities in our study, our findings suggest that Latin American cities are in very different stages of the smoking epidemic.

Our study has some limitations. We focused on cancer mortality owing to the scarcity of comprehensive incidence data from population-based registries across Latin America.^25^ This focus precludes us from disentangling the variability in incidence (linked to risk factors) and survival (linked to screening and treatment). Second, our study relies on death certificate data. Although issues such as undercounting in mortality registries have been addressed analytically, the presence of ill-defined deaths remains a challenge, along with the differential quality of death certification between and within countries, which can also differ by cancer site and SEI.^26^, ^27^, ^28^ Additionally, the time at which SEI data were collected varies between countries. Third, our ecological patterns at the city level should not be interpreted at the individual or neighbourhood levels, at which there are wide inequalities and patterns might differ. We also could not account for residual and unmeasured confounding^29^—for example due to unmeasured variables such as access to health care.

In 343 cities in nine Latin American countries, we found considerable heterogeneity in cancer mortality rates and associations with city-level socioeconomic development, and notable geographical patterns. Our results highlight the importance of considering city contexts when planning and implementing interventions to reduce the cancer burden, especially for those cancers—such as cervical and prostate cancer—that have high variability between cities. These estimates could help to plan cancer prevention and control activities through early detection, risk reduction, and management strategies centred in these cities and targeted towards socially vulnerable groups.

### Contributors

### Data sharing

Vital registration data for Brazil, Chile, Colombia, and Mexico were downloaded from publicly available repositories from statistical agencies in each country. Vital registration data for Argentina, Costa Rica, El Salvador, Panama, and Peru were obtained directly from statistical agencies in each country. The websites of these statistical agencies can be accessed via https://drexel.edu/lac/data-evidence/data-acknowledgements/. Some of the data (number of deaths in the geographical region, age, year, sex, and cause of death combined with redistributed ill-defined data and SEI data) can be made available on request at https://data.lacurbanhealth.org/.

## Declaration of interests

All authors are or have been team members of the SALURBAL project. TA is a member of the Center for Cancer Prevention and Control, funded by Fondo de Financiamiento de Centros de Investigación en Áreas Prioritarias, Fondap, Gobierno de Chile; she has received payments to her institution and to travel to SALURBAL meetings, and a consultancy fee from Organon for developing a list of women's health indicators. DS received a grant from the American Diabetes Association with support for attending meetings. JJM received grant support, paid to his institution, from the Alliance for Health Policy and Systems Research, Bloomberg Philanthropies (via the University of North Carolina at Chapel Hill School of Public Health), FONDECYT via CIENCIACTIVA/CONCYTEC, the British Council, the British Embassy, the Newton-Paulet Fund, DFID/MRC/Wellcome Global Health Trials, Fogarty International Center, Grand Challenges Canada, International Development Research Center Canada, Inter-American Institute for Global Change Research, the National Cancer Institute, the National Heart, Lung and Blood Institute, the National Institute of Mental Health, the Swiss National Science Foundation, UKRI BBSRC, UKRI EPSRC, UKRI MRC, the Wellcome Trust, and the World Diabetes Foundation and a contract from Health Action International. JJM has unpaid roles on the data safety monitoring board for the Nigeria Sodium Study; the trial steering committee for INTERACT 3; the international advisory board of the Latin American Brain Health Institute at the Universidad Adolfo Ibáñez, Chile; the consultative board of the Programa de Gastronomía, Facultad de Estudios Interdisciplinarios, Pontificia Universidad Católica del Perú; and the advisory board of the InterAmerican Heart Foundation; and is a co-chair of the Independent Group of Scientists, 2023 Global Sustainable Development Report of the United Nations; a member of the scientific expert committee of the Global Data Collaborative for CV Population Health, World Health Federation, Microsoft, and the Novartis Foundation; a member of the scientific and technical advisory committee of the Alliance for Health Policy and Systems Research, WHO; a member of the WHO technical advisory group on non-communicable disease-related research and innovation at the Noncommunicable Diseases Department, WHO; and a member of the advisory scientific committee of the Instituto de Investigación Nutricional, Peru. ML holds a National Institutes of Health (NIH)-NIDDK grant. ML, UB, and AVDR received payments to their institution from the Wellcome Trust and grants from the NIH. AVDR has roles in the NIH RECOVER Study and is a board member of the Health Effects Institute, unrelated to this study. All other authors declare no competing interests.

## Supplementary Materials

Spanish translation of the abstract

Portuguese translation of the abstract
