# Supplementary material for: Variability and social patterning of cancer mortality in 343 Latin American cities: an ecological study
Source: Lancet Glob Health. 2025 Jan 29;13(2):e268–76. doi: 10.1016/S2214-109X(24)00446-7 (PMC11782990; doi:10.1016/S2214-109X(24)00446-7)
Supplement: Spanish translation of the abstract [file mmc1.pdf]

THE LANCET
Global Health

Supplementary appendix 1

This translation in Spanish was submitted by the authors and we reproduce it as supplied. It has not been peer reviewed. *The Lancet*'s editorial processes have only been applied to the original in English, which should serve as reference for this manuscript.

Los autores nos proporcionaron esta traducción al español y la reproducimos tal como nos fue entregada. No la hemos revisado. Los procesos editoriales de *The Lancet* se han aplicado únicamente al original en inglés, que debe servir de referencia para este manuscrito.

Supplement to: Alfaro T, Martinez-Folgar K, Stern D, et al. Variability and social patterning of cancer mortality in 343 Latin American cities: an ecological study. *Lancet Glob Health* 2025; **13:** e268–76.

Antecedentes: Comprender las variaciones entre ciudades en la mortalidad por cáncer es crucial para informar las estrategias nacionales y subnacionales de prevención del cáncer. Sin embargo, los estudios a nivel de ciudad en América Latina son escasos. Como parte del proyecto Salud Urbana en América Latina (SALURBAL), nuestro objetivo fue describir la variabilidad en las tasas de mortalidad por cáncer en 343 ciudades de nueve países latinoamericanos y las asociaciones de estas tasas con el desarrollo socioeconómico a nivel de las ciudades.

Métodos: Este estudio ecológico usó datos de ciudades de Argentina, Brasil, Chile, Colombia, Costa Rica, El Salvador, Guatemala, México y Panamá. Se utilizaron datos de estadísticas vitales y de población entre el 1 de enero de 2015 al 31 de diciembre de 2019 para estimar las tasas de mortalidad por cáncer específicas por sexo y estandarizadas por edad para cada ciudad, totales y para siete localizaciones de cáncer (mama, pulmón, colorrectal, estómago, hígado, próstata y cuello uterino), y las asociaciones de estas tasas con el desarrollo socioeconómico a nivel de ciudad.

Resultados: Encontramos una amplia variabilidad en la mortalidad por cáncer por ciudad (las tasas generales de mortalidad por cáncer ajustadas por edad variaron casi tres veces), sexo y localización del cáncer. La variabilidad entre ciudades dentro del mismo país fue más alta para el cáncer de cuello uterino y de próstata. Las causas más comunes de muerte por cáncer fueron el cáncer de mama (305 ciudades) para mujeres y el cáncer de próstata (167 ciudades) y el cáncer de pulmón (132 ciudades) para hombres. El cáncer de hígado y de cuello uterino fueron la principal causa de mortalidad por cáncer en menos de diez ciudades cada uno, la mayoría en Guatemala y México. Un menor desarrollo socioeconómico a nivel de ciudad se asoció con una mayor mortalidad por cáncer de hígado, estómago, cuello uterino y próstata y una menor mortalidad por cáncer de mama, colorrectal y pulmón, variando según sexo.

Interpretación: Encontramos una considerable heterogeneidad en la mortalidad por cáncer entre ciudades, patrones geográficos y asociaciones entre las tasas de mortalidad por cáncer y el desarrollo socioeconómico. Nuestros resultados resaltan la necesidad de considerar los contextos de las ciudades en la planificación de intervenciones para reducir la mortalidad por cáncer y al guiar los futuros esfuerzos de prevención y control del cáncer en áreas urbanas dentro de la región.
