# Supplementary material for: Variability and social patterning of cancer mortality in 343 Latin American cities: an ecological study
Source: Lancet Glob Health. 2025 Jan 29;13(2):e268–76. doi: 10.1016/S2214-109X(24)00446-7 (PMC11782990; doi:10.1016/S2214-109X(24)00446-7)
Supplement: Portuguese translation of the abstract [file mmc2.pdf]

THE LANCET
Global Health

Supplementary appendix 2

This translation in Portuguese was submitted by the authors and we reproduce it as supplied. It has not been peer reviewed. *The Lancet*'s editorial processes have only been applied to the original in English, which should serve as reference for this manuscript.

Esta tradução em português foi submetida pelos autores e nós não fizemos quaisquer alterações. Esta versão não foi revista por pares. O processo editorial do The Lancet só foi aplicado à versão original em inglês, que deve servir como referência para este artigo.

Supplement to: Alfaro T, Martinez-Folgar K, Stern D, et al. Variability and social patterning of cancer mortality in 343 Latin American cities: an ecological study. *Lancet Glob Health* 2025; **13:** e268–76.

Antecedentes Compreender as variações entre cidades na mortalidade por câncer é crucial para informar estratégias nacionais e subnacionais de prevenção ao câncer. No entanto, estudos em nível de cidades, na América Latina, são escassos. Como parte do projeto Salud Urbana en América Latina (SALURBAL), nosso objetivo foi descrever a variabilidade nas taxas de mortalidade por câncer em 343 cidades de nove países latino-americanos e as associações dessas taxas com o desenvolvimento socioeconômico em nível de cidade.

Métodos Este estudo ecológico utilizou dados de cidades na Argentina, Brasil, Chile, Colômbia, Costa Rica, El Salvador, Guatemala, México e Panamá. Registros vitais e população de 1º de janeiro de 2015 a 31 de dezembro de 2019 foram usados para estimar as taxas de mortalidade total por câncer e para sete locais (mama, pulmão, colorretal, estômago, fígado, próstata e cervical), padronizadas por idade e específicas por sexo para cada cidade. Também foram avaliadas as associações dessas taxas com o desenvolvimento socioeconômico em nível de cidade.

Resultados Encontramos ampla variabilidade na mortalidade por câncer por cidade, por sexo e localidade, sendo que as taxas gerais de mortalidade ajustadas por idade variaram em quase três vezes. A variabilidade entre cidades dentro do mesmo país foi maior para os cânceres de colo de útero e próstata. As causas mais comuns foram câncer de mama em mulheres (305 cidades) e câncer de próstata (167 cidades) e de pulmão em homens (132 cidades). Câncer de fígado e de colo do útero foram as principais causas de mortalidade em menos de dez cidades, a maioria delas na Guatemala e no México. Em nível de cidades, menor desenvolvimento socioeconômico associou-se à maior mortalidade por câncer de fígado, estômago, colo de útero e próstata e à menor mortalidade por câncer de mama, colorretal e pulmão, com variações por sexo.

Interpretação Encontramos considerável heterogeneidade na mortalidade por câncer entre cidades, com distintos padrões geográficos e associações com o desenvolvimento socioeconômico. Nossos resultados destacam a necessidade de considerar os contextos urbanos ao planejar intervenções para reduzir a mortalidade por câncer e ao orientar futuros esforços de prevenção e controle do câncer em áreas urbanas da América Latina.
