# Supplementary material for: Variability and social patterning of cancer mortality in 343 Latin American cities: an ecological study
Source: Lancet Glob Health. 2025 Jan 29;13(2):e268–76. doi: 10.1016/S2214-109X(24)00446-7 (PMC11782990; doi:10.1016/S2214-109X(24)00446-7)
Supplement: Supplementary appendix 3 [file mmc3.pdf]

THE LANCET
Global Health

Supplementary appendix 3

This appendix formed part of the original submission and has been peer reviewed. We post it as supplied by the authors.

Supplement to: Alfaro T, Martinez-Folgar K, Stern D, et al. Variability and social patterning of cancer mortality in 343 Latin American cities: an ecological study. *Lancet Glob Health* 2025; **13:** e268–76.

Supplementary material

Table of contents

Table S1: List of the 343 Latin American cities included in the study, by country, with the yearly number of cancer deaths and yearly population (millions) for the 2015-2019 period. 2

Table S2: ICD-10 codes for each cancer site 8

Table S3: Population coverage of vital registration and % ill-defined deaths across the 343 cities in Latin America, by country. Numbers shown are median % [Q1-Q3] across cities. 9

Table S4: Last census availability by country and year 10

Table S5: Variability in cancer mortality rates between countries and within countries (or between cities). 11

Table S6: Five most common causes of cancer mortality in 343 cities in Latin America overall and by sex, 2015-2019. 12

Table S7: Number of cities and ranking of cancer sites by sex. 13

Table S8: Ranking of age-standardized site-specific cancer mortality rates for quartiles of SEI and the biggest city by country, stratified by sex, for seven selected sites. 14

Table S9: Ranking of age-standardized site-specific cancer mortality rates for quartiles of SEI and the biggest city by country, stratified by sex, for seven selected sites, including all the cancer sites in the position ranking. 15

Table S10: p-values for coefficients in Table 2 of main manuscript (Mortality rate ratios of cities socioeconomic development with cancer mortality rate overall and by site, stratified by sex in 343 Latin-American Cities). 16

Table S11: Interaction coefficients, 95% confidence intervals, and p-values for null hypothesis of no improvement in model fit after adding an interaction between each exposure and sex. 17

Table S12: Results from the analysis of additive interaction, including relative excess risk due to interaction (RERI) and associated 95% confidence intervals. 18

Figure S1: Age-standardized cancer mortality rates in 343 SALURBAL Cities, stratified by sex and cancer site: 2015-2019 19

Figure S2: Proportion of sum of the five (males) and six (females) selected cancer site mortality that is due to each site, by levels of the social environment index (SEI). 20

Figure S3: Association between age-adjusted cancer mortality rates and the social environment index in 343 Latin American cities. 21

Table S1: List of the 343 Latin American cities included in the study, by country, with the yearly number of cancer deaths and yearly population (millions) for the 2015-2019 period.

Country	City	Cancer Deaths	Population (millions)
Argentina	Bahia Blanca	597	0.31
Argentina	Buenos Aires	23389	15.96
Argentina	Catamarca	185	0.22
Argentina	Comodoro Rivadavia	268	0.22
Argentina	Concordia	307	0.19
Argentina	Cordoba	2615	1.71
Argentina	Corrientes	544	0.4
Argentina	Formosa	289	0.26
Argentina	Jujuy	402	0.36
Argentina	La Rioja	203	0.21
Argentina	Mar del Plata	993	0.65
Argentina	Mendoza	1674	1.2
Argentina	Neuquen-Plottier-Cipolletti	1134	0.77
Argentina	Parana	686	0.37
Argentina	Posadas	478	0.36
Argentina	Rawson-Trelew	222	0.14
Argentina	Resistencia	618	0.44
Argentina	Rio Cuarto	523	0.27
Argentina	Rio Gallegos	154	0.13
Argentina	Rosario	2714	1.45
Argentina	Salta	735	0.66
Argentina	San Carlos de Bariloche	230	0.16
Argentina	San Juan	667	0.55
Argentina	San Luis	306	0.23
Argentina	San Miguel de Tucuman-Tafi Viejo	1234	1.12
Argentina	San Nicolas de los Arroyos	280	0.15
Argentina	San Rafael	307	0.21
Argentina	Santa Fe	936	0.57
Argentina	Santa Rosa	226	0.13
Argentina	Santiago del Estero	597	0.45
Argentina	Tandil	297	0.14
Argentina	Villa Mercedes	203	0.14
Argentina	Zarate-Campana	378	0.23
Brasil	Alagoinhas	158	0.15
Brasil	Anapolis	414	0.38
Brasil	Angra dos Reis	178	0.2
Brasil	Apucarana	168	0.13
Brasil	Aracaju	845	0.94
Brasil	Aracatuba	339	0.19
Brasil	Araguaina	148	0.17
Brasil	Araguari	131	0.12
Brasil	Arapiraca	212	0.23
Brasil	Arapongas	241	0.18
Brasil	Araraquara	368	0.27
Brasil	Araras	203	0.13
Brasil	Araruama	352	0.24
Brasil	Atibaia	236	0.16
Brasil	Balneario Camboriu	269	0.21
Brasil	Barbacena	186	0.14
Brasil	Barreiras	107	0.15
Brasil	Barretos	199	0.12
Brasil	Bauru	548	0.37
Brasil	Belem	2125	2.28
Brasil	Belo Horizonte	5906	5.08
Brasil	Bento Goncalves	274	0.18
Brasil	Birigui	171	0.12
Brasil	Blumenau	677	0.56
Brasil	Boa Vista	266	0.36
Brasil	Botucatu	212	0.14
Brasil	Braganca Paulista	240	0.16
Brasil	Brasilia	3075	3.68
Brasil	Brusque	172	0.15

Brasil	Cabo Frio	433	0.35
Brasil	Cachoeiro de Itapemirim	253	0.21
Brasil	Campina Grande	621	0.49
Brasil	Campinas	3705	3.17
Brasil	Campo Grande	1031	0.88
Brasil	Campos dos Goytacazes	698	0.5
Brasil	Caraguatatuba	265	0.2
Brasil	Caruaru	351	0.35
Brasil	Cascavel	373	0.32
Brasil	Castanhal	156	0.2
Brasil	Catanduva	206	0.12
Brasil	Caxias	152	0.16
Brasil	Caxias do Sul	687	0.5
Brasil	Chapeco	221	0.21
Brasil	Conselheiro Lafaiete	146	0.13
Brasil	Criciuma	439	0.37
Brasil	Cuiaba	895	0.88
Brasil	Curitiba	3787	3.17
Brasil	Divinopolis	260	0.23
Brasil	Dourados	242	0.22
Brasil	Feira de Santana	702	0.64
Brasil	Florianopolis	1263	0.98
Brasil	Fortaleza	4022	3.68
Brasil	Foz do Iguacu	329	0.26
Brasil	Franca	420	0.35
Brasil	Garanhuns	137	0.14
Brasil	Goiania	2472	2.34
Brasil	Governador Valadares	341	0.28
Brasil	Guarapari	146	0.12
Brasil	Guarapuava	249	0.18
Brasil	Guaratingueta	368	0.27
Brasil	Ilheus	230	0.17
Brasil	Imperatriz	276	0.31
Brasil	Ipatinga	619	0.58
Brasil	Itabira	117	0.12
Brasil	Itabuna	252	0.21
Brasil	Itajai	481	0.37
Brasil	Itapetininga	193	0.16
Brasil	Jaragua do Sul	251	0.23
Brasil	Jau	234	0.15
Brasil	Jequie	202	0.16
Brasil	Ji-Parana	100	0.13
Brasil	Joao Pessoa	1198	1.13
Brasil	Joinville	698	0.61
Brasil	Juazeiro do Norte	551	0.46
Brasil	Juiz de Fora	834	0.56
Brasil	Jundiai	888	0.67
Brasil	Lages	262	0.16
Brasil	Limeira	404	0.32
Brasil	Linhares	165	0.17
Brasil	Londrina	972	0.72
Brasil	Macae	233	0.25
Brasil	Macapa	413	0.6
Brasil	Maceio	997	1.14
Brasil	Manaus	2137	2.11
Brasil	Maraba	165	0.27
Brasil	Marilia	314	0.24
Brasil	Maringa	693	0.58
Brasil	Mogi Guacu	339	0.25
Brasil	Montes Claros	431	0.4
Brasil	Mossoro	308	0.29
Brasil	Natal	1456	1.37
Brasil	Nova Friburgo	303	0.19
Brasil	Ourinhos	168	0.11
Brasil	Palmas	176	0.28
Brasil	Paranagua	165	0.15
Brasil	Parauapebas	81	0.2

Brasil	Parnaiba	171	0.15
Brasil	Parobe	259	0.18
Brasil	Passo Fundo	312	0.2
Brasil	Passos	168	0.11
Brasil	Patos de Minas	156	0.15
Brasil	Pelotas	783	0.37
Brasil	Petrolina	539	0.55
Brasil	Petropolis	532	0.31
Brasil	Piracicaba	638	0.4
Brasil	Pocos de Caldas	238	0.16
Brasil	Ponta Grossa	486	0.34
Brasil	Porto Alegre	6385	3.81
Brasil	Porto Seguro	102	0.14
Brasil	Porto Velho	450	0.53
Brasil	Pouso Alegre	174	0.15
Brasil	Presidente Prudente	355	0.25
Brasil	Recife	4271	3.75
Brasil	Resende	223	0.18
Brasil	Ribeirao Preto	927	0.69
Brasil	Rio Branco	322	0.4
Brasil	Rio Claro	317	0.23
Brasil	Rio Grande	448	0.21
Brasil	Rio Verde	149	0.22
Brasil	Rio das Ostras	192	0.18
Brasil	Rio de Janeiro	17470	12.41
Brasil	Rondonopolis	226	0.23
Brasil	Salvador	3968	3.45
Brasil	Santa Cruz do Sul	238	0.13
Brasil	Santa Maria	485	0.28
Brasil	Santarem	306	0.32
Brasil	Santos	2519	1.77
Brasil	Sao Carlos	359	0.25
Brasil	Sao Jose do Rio Preto	732	0.54
Brasil	Sao Jose dos Campos	1256	1.03
Brasil	Sao Luis	1282	1.41
Brasil	Sao Paulo	25412	21.09
Brasil	Sertaozinho	139	0.12
Brasil	Sete Lagoas	286	0.23
Brasil	Sobral	216	0.2
Brasil	Sorocaba	1047	0.82
Brasil	Tatui	164	0.12
Brasil	Taubate	641	0.52
Brasil	Teixeira de Freitas	161	0.16
Brasil	Teofilo Otoni	204	0.14
Brasil	Teresina	1061	1.02
Brasil	Teresopolis	259	0.18
Brasil	Toledo	146	0.14
Brasil	Tubarao	195	0.14
Brasil	Uberaba	427	0.33
Brasil	Uberlandia	751	0.67
Brasil	Uruguaiana	220	0.13
Brasil	Varginha	173	0.13
Brasil	Vitoria	1958	1.78
Brasil	Vitoria da Conquista	389	0.34
Brasil	Vitoria de Santo Antao	140	0.14
Brasil	Volta Redonda	834	0.58
Chile	Antofagasta	631	0.39
Chile	Arica	343	0.23
Chile	Calama	216	0.17
Chile	Chillan	371	0.23
Chile	Concepcion	1581	1.02
Chile	Copiapo	210	0.17
Chile	Curico	236	0.16
Chile	Iquique	345	0.32
Chile	La Serena-Coquimbo	717	0.47
Chile	Los Angeles	342	0.21
Chile	Osorno	282	0.17

Chile	Puerto Montt	336	0.26
Chile	Punta Arenas	227	0.14
Chile	Quillota	295	0.17
Chile	Rancagua	475	0.35
Chile	San Antonio	246	0.13
Chile	Santiago	9393	6.66
Chile	Talca	435	0.28
Chile	Temuco	557	0.37
Chile	Valdivia	271	0.17
Chile	Valparaiso-Vina del Mar	1857	0.99
Colombia	Apartado	113	0.12
Colombia	Armenia	546	0.37
Colombia	Barrancabermeja	223	0.2
Colombia	Barranquilla	2017	2.02
Colombia	Bogota	9610	8.95
Colombia	Bucaramanga	1445	1.19
Colombia	Buenaventura	199	0.3
Colombia	Buga	154	0.13
Colombia	Cali	3104	2.56
Colombia	Cartagena	908	1.06
Colombia	Cartago	193	0.13
Colombia	Cucuta	924	0.88
Colombia	Duitama	169	0.15
Colombia	Florencia	153	0.17
Colombia	Fusagasuga	194	0.14
Colombia	Girardot	182	0.14
Colombia	Ibague	675	0.52
Colombia	Manizales	735	0.49
Colombia	Medellin	5850	3.78
Colombia	Monteria	382	0.48
Colombia	Neiva	473	0.38
Colombia	Palmira	379	0.34
Colombia	Pasto	538	0.39
Colombia	Pereira	1075	0.76
Colombia	Popayan	419	0.31
Colombia	Quibdo	91	0.13
Colombia	Riohacha	136	0.18
Colombia	Santa Marta	512	0.64
Colombia	Sincelejo	225	0.27
Colombia	Sogamoso	170	0.14
Colombia	Tulua	243	0.21
Colombia	Tunja	208	0.19
Colombia	Valledupar	369	0.47
Colombia	Villavicencio	506	0.52
Colombia	Yopal	98	0.16
Costa Rica	San Jose	3048	2.56
El Salvador	San Miguel	155	0.27
El Salvador	San Salvador	1481	1.96
El Salvador	Santa Ana	244	0.27
Guatemala	Escuintla	117	0.16
Guatemala	Guatemala City	2599	3.29
Guatemala	Quetzaltenango	198	0.29
Mexico	Acapulco de Juarez	848	0.91
Mexico	Acayucan	88	0.12
Mexico	Acuna	84	0.16
Mexico	Aguascalientes	740	1.09
Mexico	Campeche	235	0.3
Mexico	Cancun	389	0.81
Mexico	Celaya	585	0.86
Mexico	Chetumal	192	0.3
Mexico	Chihuahua	928	0.95
Mexico	Chilpancingo	219	0.28
Mexico	Ciudad Valles	171	0.18
Mexico	Ciudad del Carmen	168	0.26
Mexico	Coatzacoalcos	344	0.38
Mexico	Colima	319	0.38
Mexico	Cordoba	288	0.36

Mexico	Cuauhtemoc	146	0.17
Mexico	Cuautla	351	0.49
Mexico	Cuernavaca	773	1.03
Mexico	Culiacan	797	0.94
Mexico	Delicias	128	0.15
Mexico	Durango	517	0.67
Mexico	Ensenada	390	0.51
Mexico	Fresnillo	179	0.24
Mexico	Guadalajara	3825	4.97
Mexico	Guanajuato	125	0.19
Mexico	Guaymas	187	0.22
Mexico	Hermosillo	811	0.9
Mexico	Hidalgo del Parral	115	0.11
Mexico	Iguala	105	0.15
Mexico	Irapuato	443	0.59
Mexico	Juarez	1078	1.44
Mexico	La Paz	268	0.29
Mexico	La Piedad	206	0.27
Mexico	Leon	1305	1.82
Mexico	Los Mochis	404	0.47
Mexico	Manzanillo	120	0.19
Mexico	Matamoros	373	0.53
Mexico	Mazatlan	444	0.51
Mexico	Merida	857	1.1
Mexico	Mexicali	859	1.03
Mexico	Mexico City	17038	21.55
Mexico	Minatitlan	350	0.39
Mexico	Monclova	271	0.36
Mexico	Monterrey	3817	4.74
Mexico	Morelia	871	0.94
Mexico	Navojoa	143	0.17
Mexico	Nogales	175	0.24
Mexico	Nuevo Laredo	302	0.41
Mexico	Oaxaca de Juarez	544	0.67
Mexico	Obregon	419	0.45
Mexico	Ocotlan	107	0.16
Mexico	Orizaba	352	0.47
Mexico	Pachuca de Soto	433	0.58
Mexico	Piedras Negras	121	0.2
Mexico	Playa del Carmen	80	0.22
Mexico	Poza Rica de Hidalgo	518	0.56
Mexico	Puebla de Zaragoza	2311	3.21
Mexico	Puerto Vallarta	267	0.45
Mexico	Queretaro	908	1.32
Mexico	Reynosa	539	0.79
Mexico	Rio Verde	127	0.14
Mexico	Salamanca	240	0.29
Mexico	Saltillo	674	0.95
Mexico	San Cristobal de las Casas	118	0.22
Mexico	San Francisco del Rincon	117	0.2
Mexico	San Juan Bautista Tuxtepec	149	0.17
Mexico	San Juan del Rio	176	0.28
Mexico	San Luis Potosi	878	1.16
Mexico	San Luis Rio Colorado	160	0.2
Mexico	Santo Domingo Tehuantepec	155	0.18
Mexico	Tampico	856	0.95
Mexico	Tapachula	288	0.37
Mexico	Tecoman	96	0.16
Mexico	Tehuacan	188	0.35
Mexico	Tepic	448	0.49
Mexico	Teziutlan	91	0.14
Mexico	Tianguistenco	101	0.18
Mexico	Tijuana	1355	1.92
Mexico	Tlaxcala	380	0.57
Mexico	Toluca	1216	2.28
Mexico	Torreon	1131	1.35
Mexico	Tula de Allende	214	0.32

Mexico	Tulancingo de Bravo	202	0.26
Mexico	Tuxtla Gutierrez	621	0.79
Mexico	Uriangato	85	0.12
Mexico	Uruapan	282	0.35
Mexico	Veracruz	910	0.91
Mexico	Victoria	302	0.36
Mexico	Villahermosa	552	0.85
Mexico	Xalapa	651	0.75
Mexico	Zacatecas	248	0.36
Mexico	Zamora	202	0.27
Panama	Colon	188	0.24
Panama	David	220	0.22
Panama	Panama City	1744	2.01

Footnote**:** cities selected were those with 100,000 inhabitants or more in 2010 in any of the the 11 countries of Latin America belonging to the project (countries selected based on previous collaborations), using information from different sources to create a list which were validated for each country team: The 2010 Atlas of Urban Expansion (AUE), a database of census data compiled at http://citypopulation, and Satellite imagery in Google Earth (Google, Inc.,Mountain View, California), NASA Earth Observatory Night Light Maps 2012 (NASA Worldview application, https://worldview.earthdata.nasa.gov/). Details are provided elsewhere(1).

Table S2: ICD-10 codes for each cancer site

Site	ICD-10 codes
**Overall**	C00-C97
Lung cancer	C33-C34
Colorectal cancer	C18-C21
Breast	C50
Prostate	C61
Liver	C22
Cervix	C53
Stomach	C16

Table S3: Population coverage of vital registration and % ill-defined deaths across the 343 cities in Latin America, by country. Numbers shown are median % [Q1-Q3] across cities.

Country	# Cities	% coverage	% ill-defined deaths
Argentina	33	99.96 [99.73 - 100]	5.34 [3.14 - 9.24]
Brazil	152	99.91 [98.93 - 100]	3.85 [1.68 - 7.79]
Chile	21	98.64 [95.07 - 99.37]	2.24 [1.72 - 2.49]
Colombia	35	95.72 [88.14 - 97.89]	0.94 [0.58 - 1.5]
Costa Rica	1	96.13 [96.13 - 96.13]	1.23 [1.23 - 1.23]
Guatemala	3	99.23 [99.15 - 99.61]	9.38 [8.6 - 10.42]
Mexico	92	91.16 [88.1 - 95.54]	0.75 [0.47 - 1.2]
Panama	3	97.85 [96.16 - 98.93]	2.4 [1.91 - 2.61]
El Salvador	3	96.31 [93.99 - 96.8]	30.18 [19.32 - 31.89]

Table S4: Last census availability by country and year

Country	Census year
Argentina	2010
Brazil	2010
Chile	2002
Colombia	2005
Costa Rica	2011
Guatemala	2018
Mexico	2020
Panama	2010
El Salvador	2007

Footnote: SEI, socioenvironmental index. To build SEI for Chile and Colombia census 2002 and 2005, respectively, were used.

Table S5: Variability in cancer mortality rates between countries and within countries (or between cities).

	Females		Males	
**Cancer mortality rate**	**Variability (%) between countries**	**Variability (%) within countries (1-variability between countries)**	**Variability (%) between countries**	**Variability (%) within countries (1-variability between countries)**
Overall	47.2	52.8	74.0	26.0
Lung cancer	55.5	44.5	68.3	31.7
Colorectal cancer	60.6	39.4	62.9	37.1
Breast	65.8	34.2		
Prostate			34.1	65.9
Liver	65.7	34.3	68.5	31.5
Stomach	58.3	41.7	60.2	39.8
Cervix	21.3	78.7		

Footnote: % Variability between countries is the intraclass correlation coefficient of a multilevel linear model of log(age adjusted cancer mortality rate) by each selected cancer and overall, with no predictor, and a random intercept for country. % variability within countries is the complementary (1-ICC).

Table S6: Five most common causes of cancer mortality in 343 cities in Latin America overall and by sex, 2015-2019.

**Ranking**	**1**	**2**	**3**	**4**	**5**
**Overall**	**Lung**	**Colorectal**	**Prostate**	**Stomach**	**Breast**
AAR per 100,000	13·1	9·6	9·0	8·3	7·9
AAR range	3·2 (Tianguistenco, Mexico)<br>39·9 (Antofagasta, Chile)	3·4 (Acayucan, Mexico)<br>20·6<br>(Parana, Argentina)	3·4<br>(San Miguel, El Salvador)<br>22·4<br>(Apartado, Colombia)	3·0<br>(Tecoman, Mexico)<br>23·5<br>(Popayan, Colombia)	3·7 (Parauapebas, Brazil)<br>13·8 (Corrientes, Argentina)
**Males**	**Prostate**	**Lung**	**Stomach**	**Colorectal**	**Liver**
AAR per 100,000	17·9	17·4	11·1	10·9	7·0
AAR range	6·9<br>(San Miguel, El Salvador)<br>44·9<br>(Apartado, Colombia)	3·8<br>(Tianguistenco, Mexico)<br>56·2<br>(Antofagasta, Chile)	3·7<br>(San Juan del Rio, Mexico)<br>30·9<br>(Popayan, Colombia)	3·0<br>(Caxias, Brazil)<br>26·0<br>(Parana, Argentina)	1·8<br>(Posadas, Argentina)<br>23·7<br>(Guatemala City, Guatemala)
**Females**	**Breast**	**Lung**	**Colorectal**	**Cervical**	**Stomach**
AAR Per 100,000	15·5	8·9	8·2	6·9	5·5
AAR range	7·1<br>(Araguari, Brazil)<br>27·2<br>(Corrientes, Argentina)	2·3<br>(Teziutlan, Mexico)<br>25·5<br>(Uruguaiana, Brazil)	2·5<br>(Acayucan, Mexico)<br>17·5<br>(Concordia, Argentina)	0·6<br>(Sertaozinho, Brazil)<br>22·0<br>(Manaus, Brazil)	1·8<br>(Santa Cruz do Sul, Brazil)<br>16·2<br>(Popayan, Colombia)

Footnote: AAR, age-standardized mortality rate (WHO 2000-2025 standard population).

Table S7: Number of cities and ranking of cancer sites by sex.

	Site/Rank	1	2	3	4	5	6
Females	Breast cancer	305	29	9	0	0	0
	Cervix uteri cancer	10	75	55	95	73	35
	Colon and rectum cancers	1	94	133	60	38	17
	Liver cancer	6	11	21	38	83	184
	Prostate cancer	0	0	0	0	0	0
	Stomach cancer	3	12	30	113	116	69
	Trachea, bronchus, lung cancers	18	122	95	37	33	38
Males	Breast cancer	0	0	0	0	0	324
	Cervix uteri cancer	0	0	0	0	0	19
	Colon and rectum cancers	3	39	141	114	46	0
	Liver cancer	7	15	24	57	240	0
	Prostate cancer	167	130	40	6	0	0
	Stomach cancer	34	27	94	144	44	0
	Trachea, bronchus, lung cancers	132	132	44	22	13	0

Footnote: for example, among females in 305 cities the most common cause of cancer death is breast cancer, while breast cancer is the second and third most common cause of death in 29 and 9 cities, respectively.

Table S8: Ranking of age-standardized site-specific cancer mortality rates for quartiles of SEI and the biggest city by country, stratified by sex, for seven selected sites.

Females	Breast	Cervical	Colorectal	Liver	Prostate	Stomach	Lung
Overall	1	4	3	6		5	2
Q1: Low SEI	1	2	4	5		6	3
Q2: Mid-low SEI	1	4	3	6		5	2
Q3: Mid-high SEI	1	4	2	6		5	3
Q4: High SEI	1	5	3	6		4	2
Bogota, CO	1	5	2	6		3	4
Buenos Aires, AR	1	4	3	6		5	2
Guatemala City, GT	3	4	5	1		2	6
Mexico City, MX	1	2	3	4		5	6
Panama City, PA	1	3	2	6		5	4
San Jose, CR	1	5	2	4		3	6
San Salvador, SV	1	2	5	4		3	6
Santiago, CL	1	6	3	5		4	2
Sao Paulo, BR	1	5	2	6		4	3

Males	Breast	Cervical	Colorectal	Liver	Prostate	Stomach	Lung
Overall			4	5	1	3	2
Q1: Low SEI			5	4	1	3	2
Q2: Mid-low SEI			3	5	1	4	2
Q3: Mid-high SEI			3	5	2	4	1
Q4: High SEI			3	5	2	4	1
Bogota, CO			3	5	2	1	4
Buenos Aires, AR			2	5	3	4	1
Guatemala City, GT			5	1	2	3	4
Mexico City, MX			2	5	1	4	3
Panama City, PA			3	5	1	4	2
San Jose, CR			3	5	2	1	4
San Salvador, SV			4	3	2	1	5
Santiago, CL			4	5	2	3	1
Sao Paulo, BR			3	5	2	4	1

*SEI: socioeconomic index, by quartiles (for all cities). Cut-points for the quartiles are as follows: Q1: -2.03 to -0.25, Q2: -0.25 to 0.17, Q3: 0.17 to 0.51, Q4: 0.51 to 1.09. NA: not applicable. Co: Colombia, AR: Argentina, GT: Guatemala, MX: Mexico, PA: Panama, CR Costa Rica, SV: El Salvador, CL: Chile, BR: Brazil.

Table S9: Ranking of age-standardized site-specific cancer mortality rates for quartiles of SEI and the biggest city by country, stratified by sex, for seven selected sites, including all the cancer sites in the position ranking.

**Females**	**Breast**	**Cervical**	**Colorectal**	**Liver**	**Prostate**	**Stomach**	**Lung**
Overall	1	5	4	8		6	3
Q1: Low SEI	1	3	5	6		7	4
Q2: Mid-low SEI	1	5	4	8		6	3
Q3: Mid-high SEI	1	5	3	10		6	4
Q4: High SEI	1	6	4	10		5	3
Bogota, CO	1	6	2	11		4	5
Buenos Aires, AR	1	8	4	12		10	2
Guatemala City, GT	4	5	6	1		2	8
Mexico City, MX	1	3	4	5		6	8
Panama City, PA	1	4	2	9		6	5
San Jose, CR	1	8	2	7		4	9
San Salvador, SV	1	2	6	5		4	8
Santiago, CL	1	10	3	9		6	2
Sao Paulo, BR	1	10	2	12		6	3
**Males**	**Breast**	**Cervical**	**Colorectal**	**Liver**	**Prostate**	**Stomach**	**Lung**
Overall			5	6	1	4	2
Q1: Low SEI			6	5	1	4	2
Q2: Mid-low SEI			4	6	1	5	2
Q3: Mid-high SEI			4	7	2	5	1
Q4: High SEI			3	6	2	5	1
Bogota, CO			3	9	2	1	4
Buenos Aires, AR			2	12	4	6	1
Guatemala City, GT			7	1	2	3	5
Mexico City, MX			2	6	1	5	4
Panama City, PA			3	8	1	5	2
San Jose, CR			3	6	2	1	5
San Salvador, SV			5	4	2	1	6
Santiago, CL			4	6	2	3	1
Sao Paulo, BR			3	6	2	5	1

*SEI: socioeconomic index, by quartiles(for all cities).Cut-points for the quartiles are as follows: Q1: -2.03 to -0.25, Q2: -0.25 to 0.17, Q3: 0.17 to 0.51, Q4: 0.51 to 1.09. NA: not applicable Co: Colombia, AR: Argentina, GT: Guatemala, MX: Mexico, PA: Panama, CR Costa Rica, SV: El Salvador, CL: Chile, BR: Brazil. The ranking includes all sites of cancer, so the position number exceeds the number of the seven selected sites.

Table S10: p-values for coefficients in Table 2 of main manuscript (Mortality rate ratios of cities socioeconomic development with cancer mortality rate overall and by site, stratified by sex in 343 Latin-American Cities).

Variable	Overall	Breast	Cervical	Colorectal	Liver	Stomach	Lung	Prostate
**Females**								
Q1: Low SEI	Ref.	Ref.	Ref.	Ref.	Ref.	Ref.	Ref.	NA
Q2: Mid-low SEI	0.5614	<0.0001	<0.0001	<0.0001	<0.0001	0.0004	0.1129	NA
Q3: Mid-high SEI	0.3722	<0.0001	<0.0001	<0.0001	<0.0001	<0.0001	0.0617	NA
Q4: High SEI	0.7184	<0.0001	<0.0001	<0.0001	<0.0001	<0.0001	<0.0001	NA
Males								
Q1: Low SEI	Ref.	NA	NA	Ref.	Ref.	Ref.	Ref.	Ref.
Q2: Mid-low SEI	<0.0001	NA	NA	<0.0001	<0.0001	0.0014	<0.0001	0.1196
Q3: Mid-high SEI	0.0018	NA	NA	<0.0001	<0.0001	0.0002	<0.0001	<0.0001
Q4: High SEI	<0.0001	NA	NA	<0.0001	<0.0001	<0.0001	<0.0001	<0.0001

Footnote: Age-adjusted models, random intercept for each country, fixed effect for age group. The Socioenvironmental index (SEI) corresponds to study-specific quartiles across all cities of SEI (Q1 low from -2.03 to -0.25; Q2 mid-low from -0.25 to 0.17; Q3 mid-high from 0.17 to 0.51; and Q4 high SEI from 0.51 to 1.09), which are interpreted against the reference, the lowest level (Q1 low SEI). NA: not applicable. Exponentiated coefficients and 95% CIs are available in Table 2.

Table S11: Interaction coefficients, 95% confidence intervals, and p-values for null hypothesis of no improvement in model fit after adding an interaction between each exposure and sex.

	**Overall**	**Colorectal**	**Liver**	**Stomach**	**Lung**
**RRR Q2/Mid-Low SEI (95% CI)**	1.07 (1.02-1.11)	1.05 (0.98-1.12)	1.13 (1.04-1.23)	1.05 (0.96-1.14)	1.11 (1.02-1.21)
**RRR Q3/Mid-High SEI (95% CI)**	1.08 (1.04-1.13)	1.1 (1.03-1.18)	1.26 (1.15-1.37)	1.16 (1.07-1.26)	1.15 (1.06-1.25)
**RRR Q4/High SEI (95% CI)**	1.11 (1.06-1.16)	1.13 (1.05-1.2)	1.24 (1.14-1.35)	1.19 (1.09-1.29)	1.11 (1.02-1.21)
Joint p-value (for all 3 RRRs)	<0.0001	0.0017	<0.0001	<0.0001	0.0078

Footnote: p-value was calculated using a log likelihood ratio test of nested models m1 and m2, where m1: pooled model similar to main model but with both sexes and a fixed effect for sex; m2: m1+ interaction term between exposure and sex. The relative rate ratio (RRR) comes from exponentiating each interaction coefficient in m2. Prostate, breast, and cervical cancer are not included as they have very low counts in one of the sexes.

Table S12: Results from the analysis of additive interaction, including relative excess risk due to interaction (RERI) and associated 95% confidence intervals.

	Higher SES : Higher Cancer Mortality	Higher SES: Lower Cancer Mortality
RERI > 0 (stronger association in males)	Lung (RERI=0.15 [0.07 to 0.23])<br>Colorectal (RERI=0.17 [0.11 to 0.22])	
RERI < 0 (stronger association in females)		Overall (RERI=-0.06 [-0.1 to -0.03])<br>Liver (RERI=-0.14 [-0.23 to -0.04])<br>Stomach (RERI=-0.12 [-0.22 to -0.03])

Footnote: In these models, SEI was binary (dichotomized at the median). The Relative Excess Risk due to Interaction (RERI) comes from the formula RERI=RR11-RR10-RR01+1, where RR11 is the RR comparing males in the high (or low) SEI category vs females in the referent SEI category (low or high), RR10 is the RR comparing males to females living in low or high SEI areas, and RR01 is the RR comparing the high (or low) category to the referent SEI category (low or high) among females. 95% confidence intervals were calculated using the delta method. Whether the high or low SEI category was used as a reference depended on the pattern of the SEI/Cancer association (e.g., for High SEI : High Cancer associations, the low SEI group was used as the reference).

Figure S1: Age-standardized cancer mortality rates in 343 SALURBAL Cities, stratified by sex and cancer site: 2015-2019

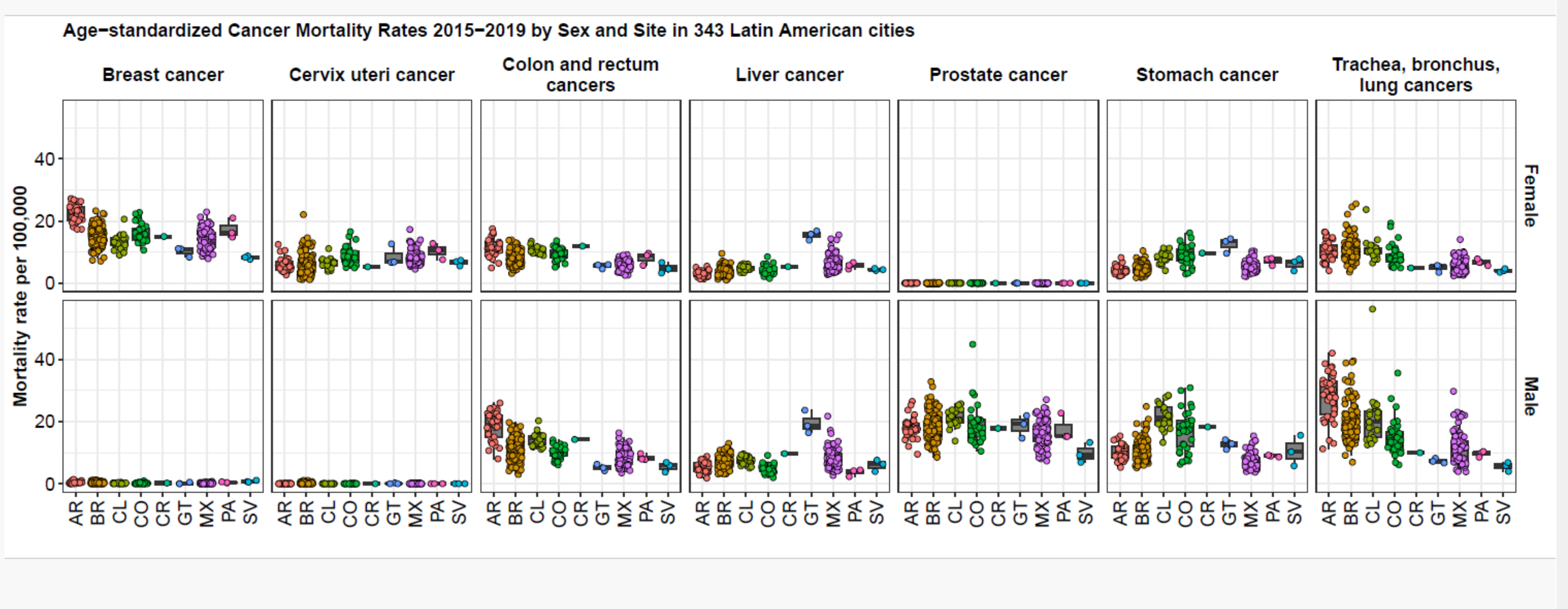

Footnote: AR, Argentina; BR, Brazil; CL, Chile; CO, Colombia; CR, Costa Rica; GT, Guatemala; MX, Mexico; PA, Panama; SV, El Salvador.This plot shows all data points, regardless of whether they are outliers or not, and the corresponding box-and-whisker plot without outliers.

Figure S2: Proportion of sum of the five (males) and six (females) selected cancer site mortality that is due to each site, by levels of the social environment index (SEI).

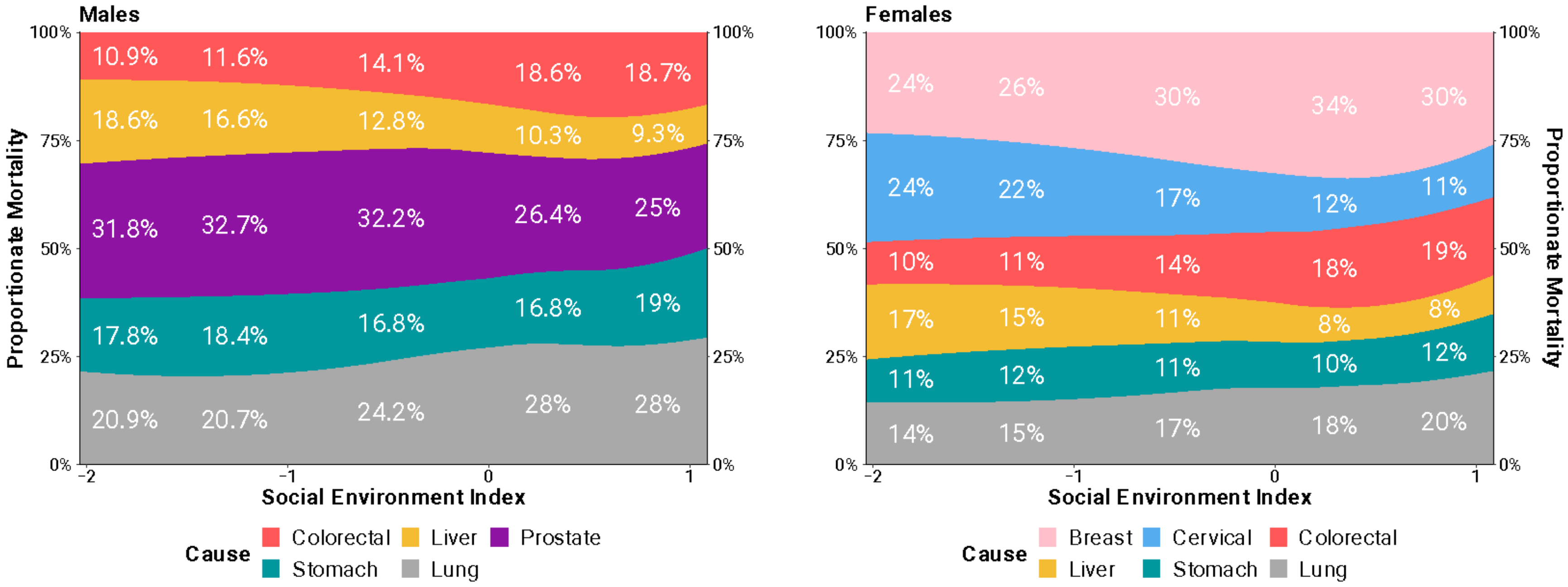

Figure S3: Association between age-adjusted cancer mortality rates and the social environment index in 343 Latin American cities.

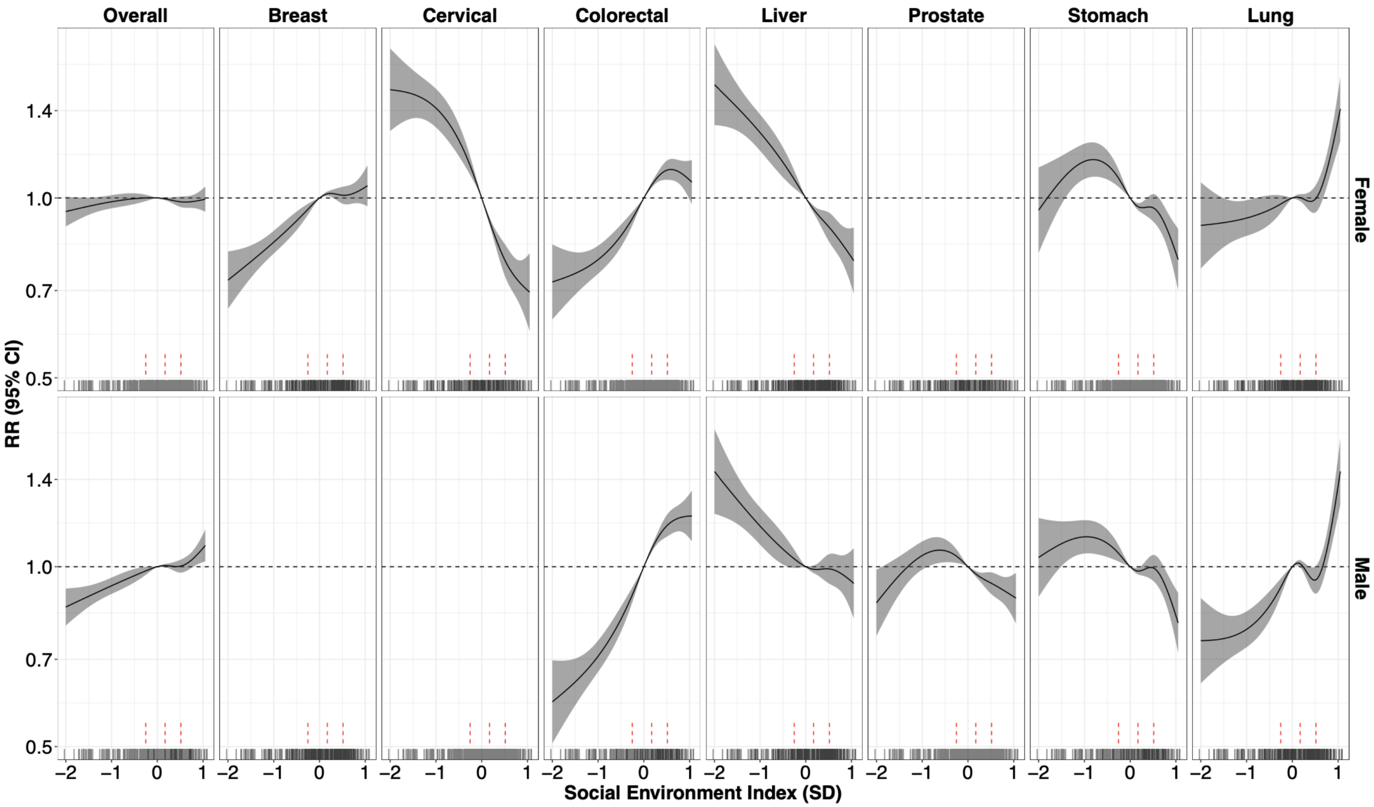

Footnote: Age-adjusted negative binomial models, random intercept for each country, fixed effect for age group, and restricted cubic splines for the social environment index with 3 knots at the 25th, 50th, and 75th percentiles. Black marks on the x-axis represent cities with a given value of the social environment index. The three red dashed lines represent the location of the knots.

References

1. Quistberg DA, Diez Roux AV, Bilal U, Moore K, Ortigoza A, Rodriguez DA, et al. Building a Data Platform for Cross-Country Urban Health Studies: the SALURBAL Study. Journal of Urban Health. 2019;96(2).
